# Morphological description of *Laevicaulisstuhlmanni* (Simroth, 1895) (Pulmonata, Veronicellidae) from Egypt

**DOI:** 10.3897/BDJ.10.e85495

**Published:** 2022-10-07

**Authors:** Reham Fathey Ali, David G Robinson, Fabio Liberto

**Affiliations:** 1 Faculty of Organic Agriculture, Heliopolis University, Cairo, Egypt Faculty of Organic Agriculture, Heliopolis University Cairo Egypt; 2 Faculty of Agriculture, Cairo University, Giza, Egypt Faculty of Agriculture, Cairo University Giza Egypt; 3 USDA APHIS PPQ National Malacology Laboratory, Philadelphia, Pennsylvania, United States of America USDA APHIS PPQ National Malacology Laboratory Philadelphia, Pennsylvania United States of America; 4 Via del Giubileo Magno 93, 90015, Cefalù, Italy Via del Giubileo Magno 93, 90015 Cefalù Italy

**Keywords:** slug, veronicellid, taxonomy, morphology, genitalia, north-eastern Africa

## Abstract

**Background:**

Terrestrial slugs and snails are increasingly becoming serious pests of agricultural, horticultural and ornamental plants in Egypt, resulting in major economic losses.

**New information:**

This paper provides a detailed morphological and anatomical description of the veronicellid slug *Laevicaulisstuhlmanni* (Simroth, 1895) that has been recently recorded from Egypt. This population from Egypt is compared with *Laevicaulis* populations recently reported and described from Libya as *L.striatus* (Simroth, 1896) and with available data in literature. Some notes and a distribution map of the veronicellids introduced in north-eastern Africa are provided.

## Introduction

Terrestrial slugs are reported as abundant and common pests that feed on crops in agricultural areas and ornamental plants in nurseries and gardens in Egypt. These pests contribute to increasing losses of the agricultural production and decreasing economic value of ornamental plants (South 1992, Barker 2002, Howlett 2012).

Recently, a number of terrestrial slugs have been recorded in Egyptian nurseries and gardens, such as *Derocerasreticulatum* (O.F. Müller, 1774) (Agriolimacidae) (Rady et al. 2014, Shahawy 2019), *Deroceraslaeve* (O.F. Müller, 1774) (Agriolimacidae) (Obuid-Allah et al. 2008, Mohamed and Ali 2011, Abou Senna et al. 2016), *Limacusflavus* (Linnaeus, 1758) (Limacidae) (Gamil 2013, Mohamed and Ali 2013), *Ambigolimaxvalentianus* (A. Férussac, 1821) (Limacidae) (Ali and Robinson 2020) and *Laevicaulisalte* (A. Férussac, 1821) (Veronicellidae) (Ali and Robinson 2020, Ali and Robinson 2022).

The Veronicellidae is a systellommatophoran mollusc family that occurs mainly in the tropical and subtropical region in Central and South America (Naranjo-García and Thomé 2007, Robinson et al. 2009, Santin and Miquel 2015, Oliveira Rocha 2019), Southern and Southeast Asia, Hawaii (Kim et al. 2016), Indian Ocean Islands (Herbert and Kilburn 2004) and Sub-Saharan Africa including the Democratic Republic of Congo (formerly Zaire), Malawi, South Africa and Tanzania (Forcart 1953, Herbert and Kilburn 2004, Rowson B et al. 2017).

The genus *Laevicaulis* Simroth, 1913 is native to Sub-Saharan Africa. However, *Laevicaulisalte* (A. Férussac, 1822) has been introduced by human activity to other areas of the world, where it has become abundant and causes considerable damage to agricultural crops (Gomes and Thomé 2004). Recently, three *Laevicaulis* species have been reported in agricultural fields and gardens in north-eastern Africa: *L.alte* and *L.stuhlmanni* (Simroth, 1895) from Egypt (Ali 2017a, Ali 2017b, Ali and Robinson 2020, Ali and Robinson 2022) and *Laevicaulisstriatus* (Simroth, 1896) from Libya (Liberto et al. 2021). In this paper, the external morphology and genitalia of *L.stuhlmanni* from Egypt are described in detail, comparing them whith *L.striatus* from Libya and with the available literature data.

## Materials and methods

Specimens of *Laevicaulisstuhlmanni* were collected from the indoor ornamental plants garden of a hotel located in El Gezira Street, on Gezira Island, El Zamalek district, Cairo, Cairo Governorate, Egypt (30°03’27.4” N 31°13’28.1” E) in April 2016 in the late evening when they are typically more active. The specimens were collected from humid areas, such as near irrigation tubes, close to water faucets, under leaf litter and stones. The collected samples were transferred to the Malacology Laboratory, Department of Zoology and Agricultural Nematology, Faculty of Agriculture, Cairo University in Giza. The slugs were drowned in water and then preserved in 85% ethanol and they were dissected as described in Gomes et al. (2013). The specimens were measured by using digital caliper for: total body length, maximum body width, foot length, foot width, width of right and left hyponota and the distance of the female genital pore from the posterior end of the slug body (n = 15), recording the external features and characters that are important in identifying the species. These include the position of the female genital pore, width of the foot relative to the hyponotum, in addition to the position and the shape of the anus.

The reproductive system was removed using ocular surgical scissors, forceps and pins. In the anatomical description, proximal denotes the part which is closest to the gonad and distal the part which is closest to the female genital pores. All the specimens were studied by a Nikon SMZ1500 stereomicroscope. Some organs of genitalia were measured by a digital caliper. Photographs were taken with a Nikon digital Sight DS-Fil camera attached to the Nikon SMZ155 stereomicroscope. Some drawings, based on these photographs, were made using CorelDraw X5.

## Taxon treatments

### Laevicaulis stuhlmanni

(Simroth, 1895)

4E82F6BD-9656-5FAF-95A9-E70694DCB90F

https://www.gbif.org/species/11057552

https://www.molluscabase.org/aphia.php?p=taxdetails&id=1255847

https://eol.org/pages/52584426

https://en.wikipedia.org/wiki/Laevicaulis_stuhlmanni

https://www.molluscabase.org/aphia.php?p=taxdetails&id=1335505

**Synonymy**
*Vaginula stuhlmanni* Simroth, 1895 (original combination)
Eleutherocaulis stuhlmanni (Simroth, 1895)
*Vaginula schnitzleri* Simroth, 1895
Vaginula aequatorialis Simroth, 1896
*Vaginula brauni* Simroth, 1913
Laevicaulis stuhlmanni aegypti Ali and Robinson, 2017 (*nomen nudum*)

#### Materials

- **Type status:** Other material. **Occurrence:** occurrenceID: 8C0A5648-A953-5B9F-B900-5D4F0307EB8A; catalogNumber: USDA 144250; occurrenceRemarks: found on grass in Marriot Hotel in Cairo; recordedBy: Reham Fathey Ali; individualCount: 25; sex: hermaphrodite; lifeStage: juvenile and adults; preparations: whole animal (ETOH); **Taxon:** scientificNameID: urn:lsid:marinespecies.org:taxname:1255847; scientificName: *Laevicaulisstuhlmanni* (Simroth, 1895); kingdom: Mollusca; class: Gastropoda; family: Veronicellidae; **Location:** continent: Africa; country: Egypt; stateProvince: El Gezira Street, on Gezira Island, El Zamalek district; county: Egypt; **Event:** samplingProtocol: collecting by hand and observation; year: 2006; month: 4; day: 15; habitat: garden; eventRemarks: collecting at late hours of night in activity periods; **Record Level:** institutionCode: USDA – USDA APHIS National Malacology Collection, Academy of Natural Sciences, Philadelphia, Pennsylvania, USA; collectionCode: "Terrestrial slugs"; basisOfRecord: PreservedSpecimen

#### Description

**General description of external morphology**

**Figure 1.**
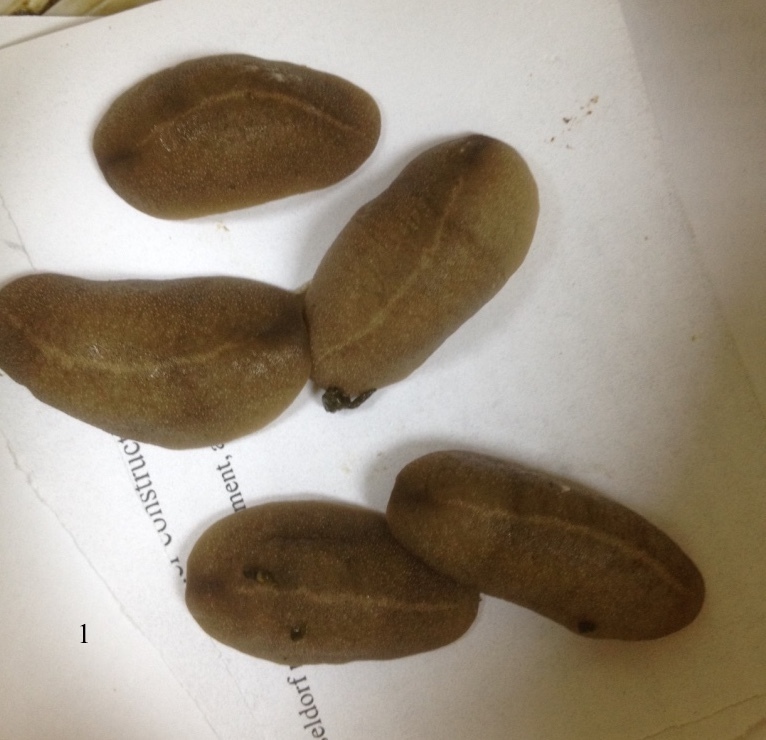
*Laevicaulisstuhlmanni* (Simroth, 1895) Gezira Island, Cairo, Egypt; external morphology and colouration.

**Figure 2.**
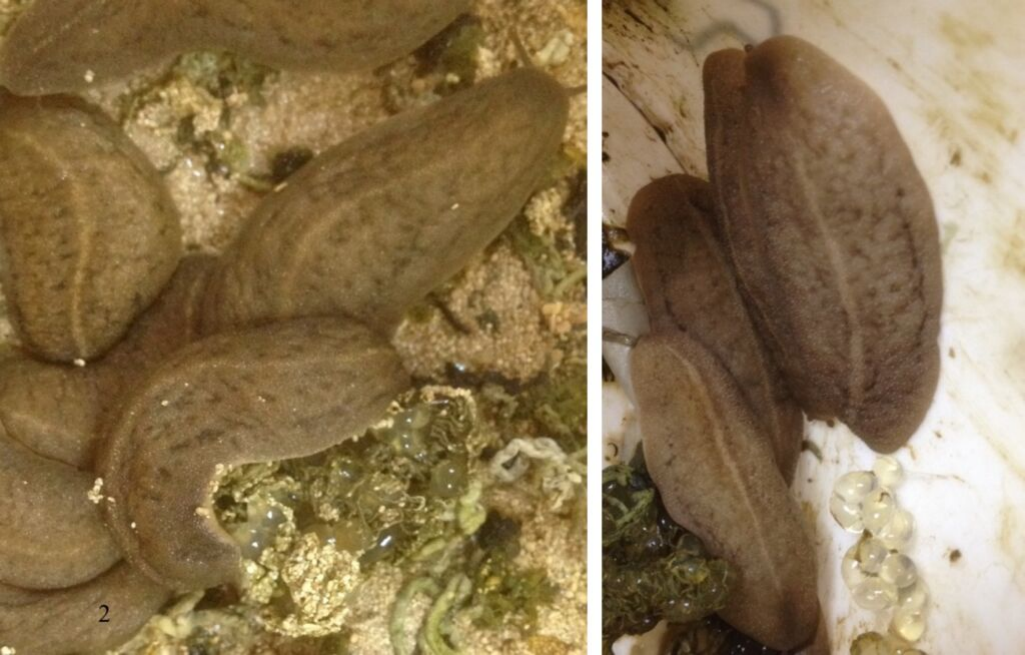
*Laevicaulisstuhlmanni* (Simroth, 1895) Gezira Island, Cairo, Egypt.

The slug has a dorsal-ventrally flattened body; the notum has a dark brown background, with a light longitudinal colour band running down the centre; in some specimens, it is reduced to punctation (Fig. 1). Some specimens have a darker band on both sides of the notum, these bands being connected together on the head and in the back and could be thick or thin or as rows of dark spots (Figs 1, 2). The hyponata are uniform light brown. The head has two pairs of tentacles that are hidden under the notum, the first (lower) shorter chemotactic pair and a longer (upper) pair of ocular tentacles. The adult specimens of *Laevicaulisstuhlmanni* preserved in alcohol have been measured (n = 15): mean length 44 mm (range: 36–52 mm), mean width 16 mm (range: 12–19 mm), mean foot length 38 mm (range: 23–48 mm), mean foot width 6 mm (range: 4-9 mm), mean width of right hyponota 5 mm (range: 3–7 mm), mean width of left hyponota 5 mm (range: 3-7 mm).

#### Diagnosis

**Figure 3.**
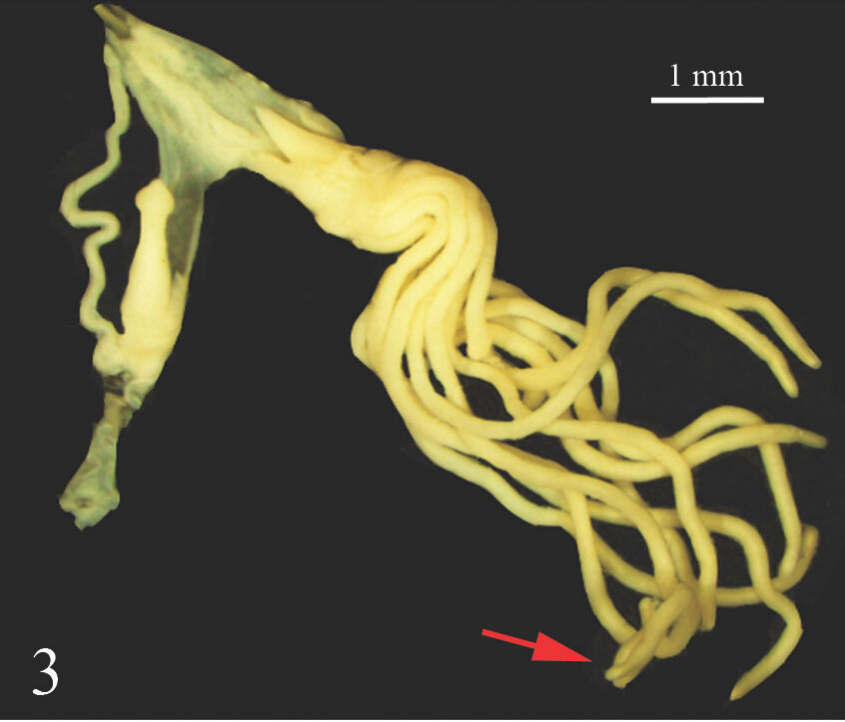
*Laevicaulisstuhlmanni* (Simroth, 1895) Gezira Island, Cairo, Egypt, transverse dissecting view of the phallic sheath; the arrow points to tubule branched into two smaller tubules.

**Figure 4.**
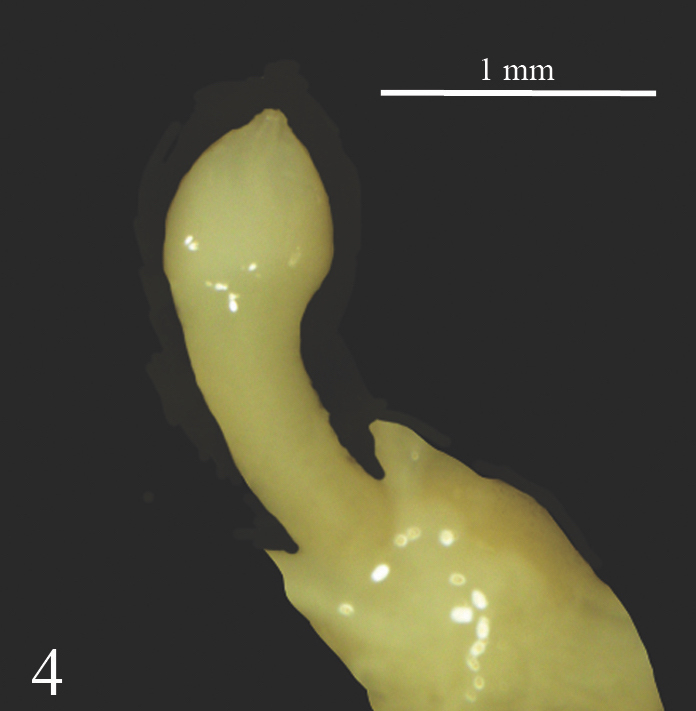
*Laevicaulisstuhlmanni* (Simroth, 1895) Gezira Island, Cairo, Egypt, phallus.

**Figure 5.**
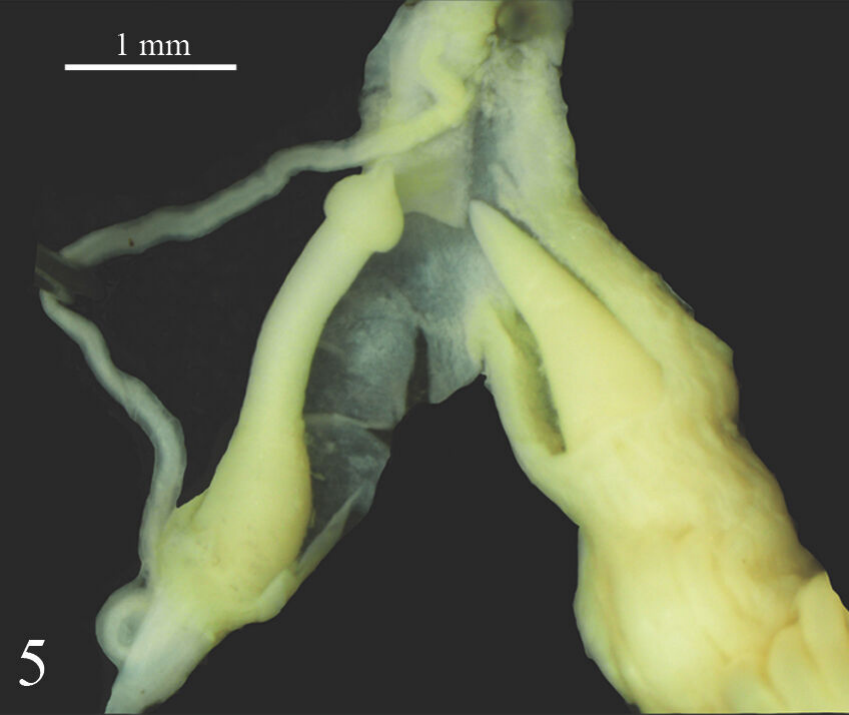
*Laevicaulisstuhlmanni* (Simroth, 1895) Gezira Island, Cairo, Egypt, the verge with contracted distal part, the phallus gland tip is at the same level as the phallus tip.

**Figure 6.**
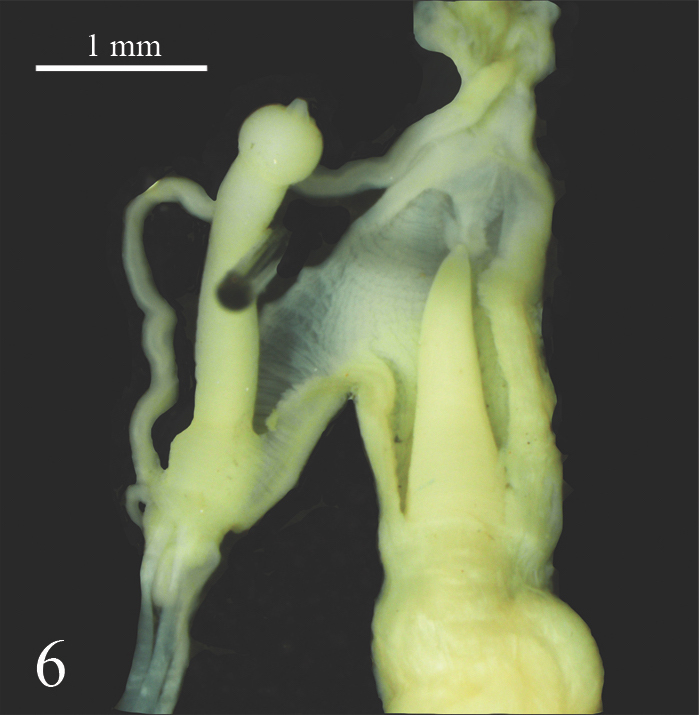
*Laevicaulisstuhlmanni* (Simroth, 1895) Gezira Island, Cairo, Egypt, the verge with erected distal part, the phallic gland tip is not at the same level as the phallus tip.

**Figure 7.**
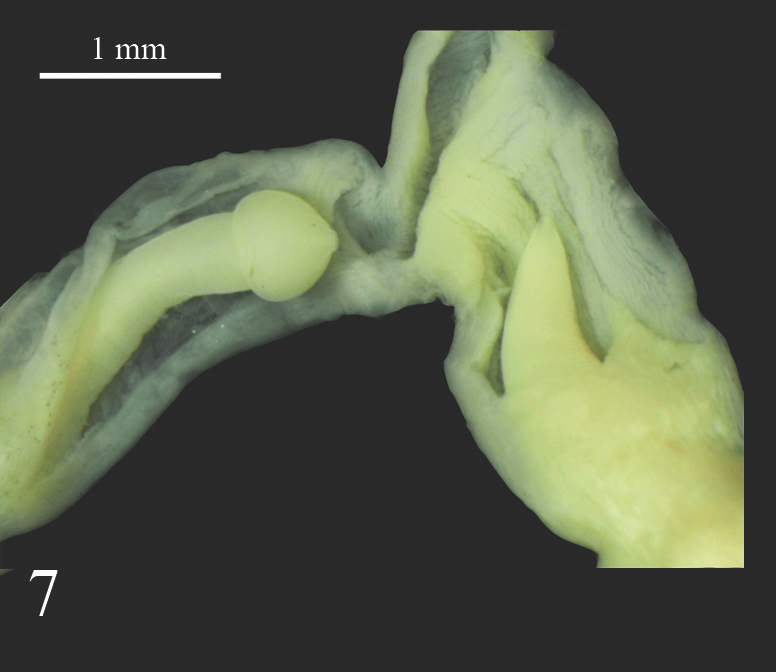
*Laevicaulisstuhlmanni* (Simroth, 1895), transverse dissecting view of the phallic sheath; on the base of papilla, there are four to five thin lines of wrinkles.

**Genitalia description**:

The female genital pore is located on the right hyponotum, posterior to the middle of the total length and the male genital pore opens in the anterior portion just below the right ocular tentacle. The anterior male genital complex is composed of two elements, the phallus *sensu stricto* and the phallic gland, both enclosed in a muscular sheath sharing a common atrium that opens through the genital pore. The phallus is slender (Figs 3, 4) and has a sub-distal annular swelling. In some specimens, the phallus is less slender, with a distal swelling, caused by the invagination of the distal part. The phallic gland is composed of a conical papilla that arises from a vase-shaped base. On the proximal part, there are a number digitiform tubules, numbering from ten to seventeen, averaging 12.5 ± 1.8 tubules. In three specimens, it was noticed that one of the tubules branched into two smaller tubules (Fig. 3). The papilla gland is at the same level as the phallus papilla tip in some samples or distally forward (Figs 5, 6). However, in two specimens, the phallus papilla stalk was distally more forward than the gland papilla or not at the same level (Fig. 7). The papilla of the phallic gland is pointed conical and different in length and width relative to its base in each individual; at the base of the papilla, typically four to five thin lines of wrinkles can be seen, possibly produced by the contraction and expansion of the papilla. Some specimens have up to seven to twelve thin lines of wrinkles (Fig. 7). The posterior genitalia are characterised by a more or less oval hermaphroditic gonad (ovotestis) made up of rounded acini; from it arises a large convoluted hermaphroditic duct that connects to the albumen gland, the latter being conical and very large. The vas deferens can be divided into four parts: the proximal posterior vas deferens (from the fertilisation complex to the prostatic gland), the distal posterior vas deferens (from the prostate to the ligament duct), the middle vas deferens (from the ligament duct, in anterior direction inside the integument) and the anterior vas deferens (from the exit of the tegument to the phallus). The prostatic gland is dark yellow to pale coffee colour with a smooth surface; the ligament duct is a short duct that connects the middle vas deferens to the short duct of the bursa copulatrix; the bursa copulatrix is large and oval (Fig. 8).

#### Distribution

**Figure 9.**
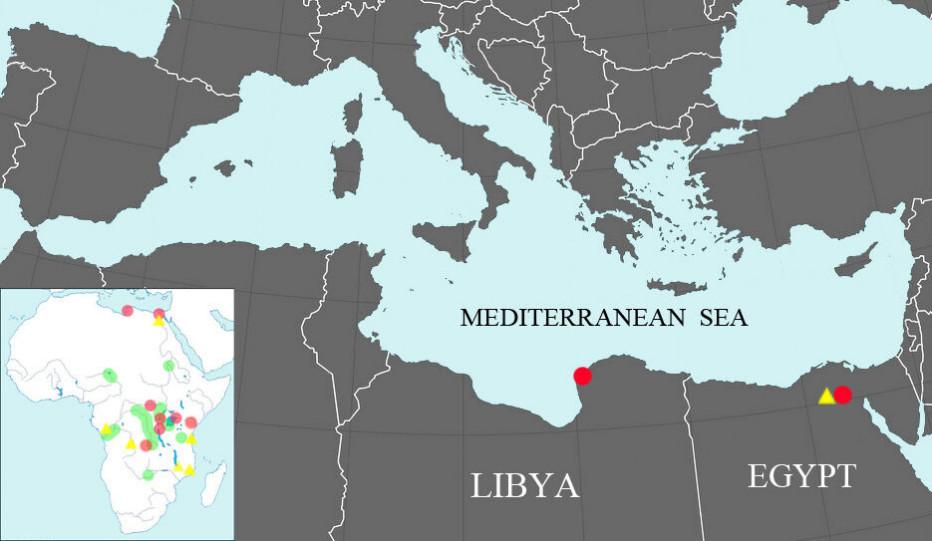
Distribution map of *Laevicaulisstuhlmanni* (red dots), *Laevicaulisalte* (yellow triangle) and *Laevicaulisstriatus* (green dots) in Africa.

*Laevicaulisstuhlmanni* was first collected from the indoor ornamental plants garden of a hotel located in El Gezira Street, on Gezira Island, Cairo, El Zamalek District, Cairo, Egypt (30°03’27.4”N 31°13’28.1”E). There have been subsequent unconfirmed reports from anthropochorus environments elsewhere in Egypt. The distribution map of *Laevicaulisstuhlmanni, Laevicaulisalte* and *Laevicaulisstriatus* is illustrated in Africa (Fig. 9).

#### Biology

The species *Laevicaulisstuhlmanni* was reported for the first time in Egypt under the *nomen nudum L.stuhlmanniaegypti* (Ali 2017a, Ali 2017b) and the biological attributes, such as life cycle, pre-oviposition period, oviposition period, post-oviposition period, reproductive output, incubation period, generation period and life span, were documented and described.

**Figure 10.**
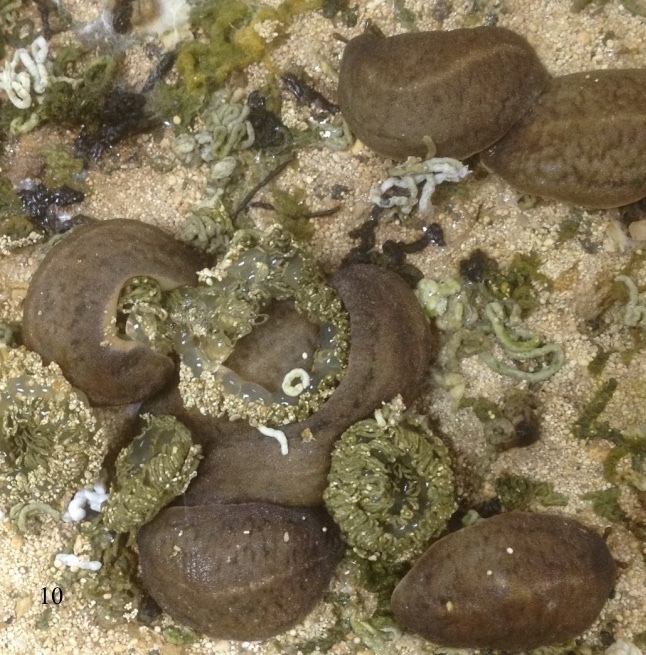
*Laevicaulisstuhlmanni* (Simroth, 1895) Gezira Island, Cairo, Egypt; the photos include the egg masses of this species. The eggs are joined together by a thin interconnecting thread that forming spiral-like egg mass.

**Figure 11.**
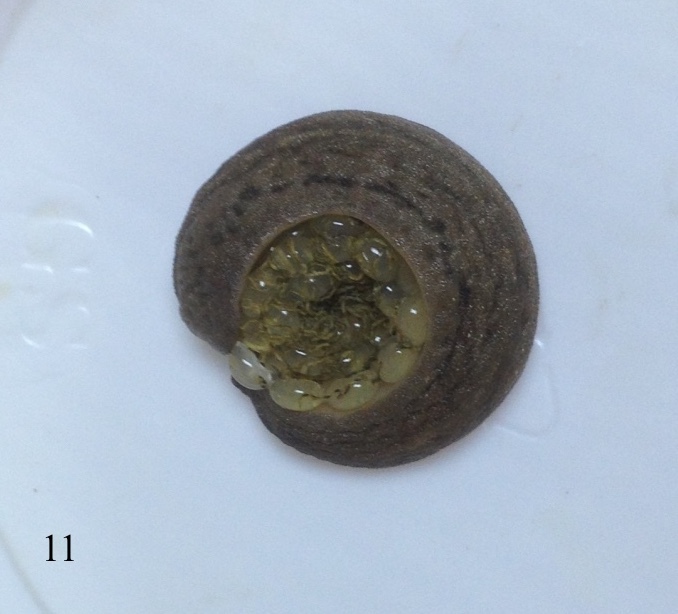
*Laevicaulisstuhlmanni* (Simroth, 1895) Gezira Island, Cairo, Egypt; the species during the egg-laying process under laboratory conditions.

**Figure 12.**
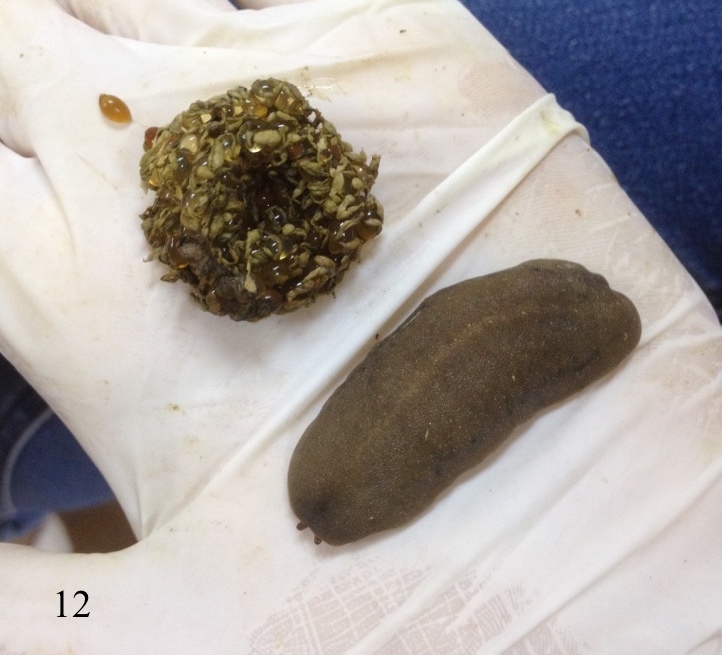
*Laevicaulisstuhlmanni* (Simroth, 1895) Gezira Island, Cairo, Egypt; the species deposited eggs that are darker in colour ready to hatch and covered with a distinct faecal ribbon on the top of the eggs.

As in all veronicellid species, the slug is a hermaphrodite species that possessing both male and female genitalia and self-fertilisation can occur. However, mating or cross-fertilisation was not observed for this species under laboratory conditions. The reproductive season of this species starts in March, when the seasonal temperature rises and lasts until November (Ali 2017b). The incubation period for the eggs to hatch averaged 15 days with ranges from 10 to 19 days at a temperature of 29 to 31°C and a humidity between 52% and 64%. *Laevicaulisstuhlmanni* reaches sexual maturity after an average of 86 days with ranges 53 to 115 days after hatching, depending on the surrounding conditions. Total life span averaged 155 days with ranges between 127 to 188 days. The oviposition period averaged 46 days with ranges one to 74 days; each slug can produce around five egg clutches ranging from one to twelve egg clutches/slug with an average of 47 eggs/clutch (range 10 to 120 egg/clutch) under laboratory conditions. Generation period averaged 102 days with ranges 69 to 127 days (Ali 2017b). This species laid multiple egg masses during its activity months. In Figs 10, 11, 12, the eggs are oval to spherical elongate shape and translucent. The eggs are joined together by a thin interconnecting thread producing a gradually spiral-like egg mass with distinct faecal pellets ribbon deposited on the top of the eggs.

#### Taxon discussion

*Laevicaulisstuhlmanni* is a native slug of Eastern and Central Africa (Congo, Rwanda, Burundi, Kenya, Tanzania) (Forcart 1953). Veronicellids have been recorded throughout the tropics on a number of ornamental plants including flower beds and seedlings causing significant economic losses (Robinson and Hollingsworth 2005 , Gomes et al. 2013). It is a phytophagous species and often occurs in large numbers beneath decaying vegetation in its natural habitat; however, through this study and from other growers and farmers’ observations, the species was also found in commercial plantations. *L.stuhlmanni* is usually active during the night, while it is buried in the ground during the day (Ali 2017b). It is a hermaphrodite and member of a genus that includes some widespread and invasive pests, such as *Laevicaulisalte* (Raut 1999, Das and Parida 2015).

#### Taxonomic remarks

The validity of *Laevicaulis* Simroth, 1913 as the objective senior synonym of *Eleutherocaulis* Simroth, 1913 was recently confirmed by Sajan and Tripathy (2021). The type species, in both cases by subsequent designation, is *Vaginuluscomorensis* Fischer, 1883, a junior synonym of *Vaginulusalte* A. Férussac, 1822.

The systematics of the *Laevicaulis* species are uncertain, due to the description of several taxa in the second half of the 1800s, based only on external characters such as body colour and body measurements of the slug or on inadequate descriptions of genitalia. Simroth (1895) described a subspecies *Laevicaulisstuhlmanniatrolimbatus*, but its validity is questionable.

*Laevicaulisstriatus* and *L.stuhlmanni* have similar female genitalia and phallic glands. However, the phallus in the two species reportedly has a different apex. In Simroth 1895: 62, the original description of *L.stuhlmanni* describes a cylindrical phallus, slightly tapered distally, with a flat terminal disc, in the middle of which the sperm duct opens. The descriptions and figures of *L.stuhlmanni* in Simroth (1896: 18, Pl. 3, figs. 6 B-C) and the synonym *Vaginulaaequatorialis*) and those of Forcart L (1953: 74-76, Pl. 5, figs. 1 A-E) are consistent with the original description of *L.stuhlmanni*. However, part of Forcart's description of *L.stuhlmanni* (Forcart 1953: 75, Pl. 4 figs. 10 A-B) is not consistent with *L.stuhlmanni*'s original description, while it fits well with *L.striatus* in the same paper (Forcart 1953). Simroth (1896) described the new species *Vaginulastriata*, based on external features of sexually immature specimens. Forcart (1953: 79-86, Pl. 5, figs. 3 A and C) reviewed *L.striatus*, based on adult specimens sampled in the type locality. These specimens are characterised by the phallus with a subdistal annular swelling. The important character given for *L.stuhlmanni* is that it has a phallus with a flat terminal disc, while *L.striatus* has a phallus with a subdistal annular swelling. Based on these data, Liberto et al. (2021) classified the *Laevicaulis* population from Libya as *L.striatus*.

However, as shown by Colosi (1927) for *Laevicaulissomalicus* (Colosi 1927) and as evidenced by Forcart (1953) (P1. 4, fig. 10; P1. 5, figs. 1, 3) for *L.stuhlmanni* and *L.striatus*, the length of the phallus and the shape of the phallic apex are variable according to the contraction or erection of the phallus; therefore, it cannot be excluded that *L.striatus* is a synonym of *L.stuhlmanni*. Pending a modern taxonomic review involving a molecular analysis of the *stuhlmanni/striatus* group, the authors attribute the population examined here and the one reported from Libya (as *Eleutherocaulisstriatus* in Liberto et al. (2021) to the senior taxon *L.stuhlmanni* (Simroth, 1895). The length of the phallus stalk of *L.stuhlmanni* in Forcart (1953) (when not contracted) is similar to that measured in the population from Cairo (Egypt) i.e. one or one and a half times the length of the papilla of the phallic gland, whereas in the populations from Benghazi (Libya), it is longer, i.e. two and a half times. The *L.stuhlmanni* populations described by Forcart (1953) have a variable external colour: greyish, reddish, yellow, brown or blackish-brown, the sole is uniformly blackish and always much darker than the hyponota or light with blackish margin ("subspecies" *atrolimabratus*). *L.stuhlmanni* from Egypt and Libya has an external colouration more similar to that described by Forcart (1953) for *L.striatus*: notum reddish-brown, yellow or greenish-brown unicoloured or with a median stripe, flanked on each side by two rows of irregular dark spots or stripes; hyponota and foot lighter than the notum.

Recently, Ali and Robinson (2020) and Ali and Robinson (2022) recorded *Laevicaulisalte* from Abo Rawash, Giza, Egypt. *L.alte* is distinguished from *L.stuhlmanni* by having a very dark notum with a pale thin well-defined white or creamy line on the dorsal surface, running the length of the body; the lighter hyponota have a creamy or grey colour; both surfaces minutely granular; the phallus with a sub-basal annular swelling (Grimpe and Hoffmann 1925, Benthem-Jutting 1952,Forcart 1953, Brodie and Barker 2012, Prakash et al. 2015).

## Discussion

The Veronicellidae is a common phytophagous family, occurring mainly in the tropical and subtropical areas of America, Asia and Africa (Baker 1926, Aguayo 1965, Naranjo-García and Thomé 2007, Robinson et al. 2009). More specifically, these slugs are known from Central and South America (Thomé 1993, Santin and Miquel 2015, Oliveira Rocha 2019), Sub-Saharan Africa including the Democratic Republic of Congo (formerly Zaire), Malawi, South Africa and Tanzania (Forcart 1953, Herbert and Kilburn 2004, Rowson B et al. 2017), Southern and Southeast Asia, Hawaii (Hata et al. 1997, Kim et al. 2016) and Indian Ocean Islands (Herbert and Kilburn 2004), with a first record finding the slug species *Semperulawallacei* (Issel, 1874) (Veronicellidae) in Japan (Hirano et al. 2018). These slugs are hermaphroditic and lack an internal shell or calcareous particles (Gude 1914, Runham and Hunter 1970, Barker 2001).

Members of the Veronicellidae are potentially very serious agricultural pests causing great economic loss for important field crops, being voracious plant feeders and as vectors of parasites affecting humans and livestock, as well as carriers for plant diseases (Gomes and Thomé 2001). In Central and South America, some species are intermediate hosts of parasitic nematodes, such as *Angiostrongyluscostaricensis* causing abdominal angiostrongyliasis and the rat lungworm *Angiostrongyluscantonensis* that is the etiologic agent of eosinophilic meningitis in the Pacific Islands (Caldeira et al. 2007). The emergence of this new reported slug pest is due to unsuccessful quarantine barriers and plant protection regulations. The authors are establishing an initial wide database of well-identified invasive gastropods that are found in some Egyptian fields with more new reported species that have been added to the list as potential agricultural pests (Ali and Robinson 2020). The present work recognises *Laevicaulisstuhlmanni* as a species recently introduced to Egypt with a potentially rapid expansion in cultivated fields and irrigated gardens, with consequent damage to field crops and ornamental plants.

## Supplementary Material

XML Treatment for Laevicaulis stuhlmanni

## Figures and Tables

**Figure 8a.**
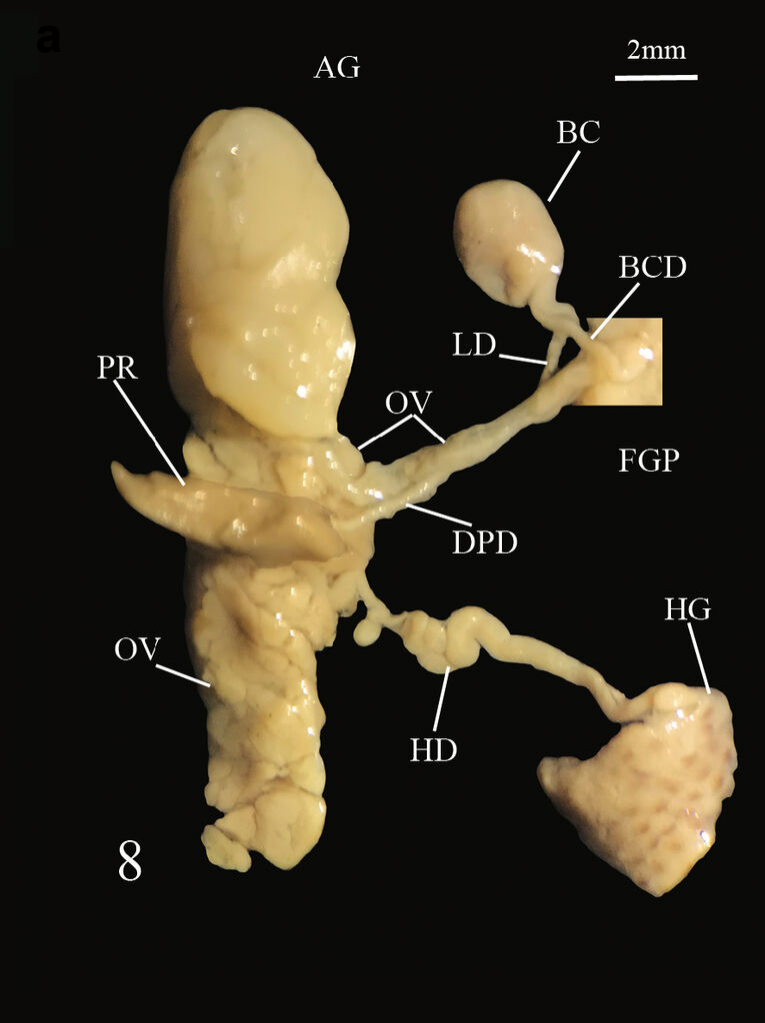
Photograph of female reproductive system.

**Figure 8b.**
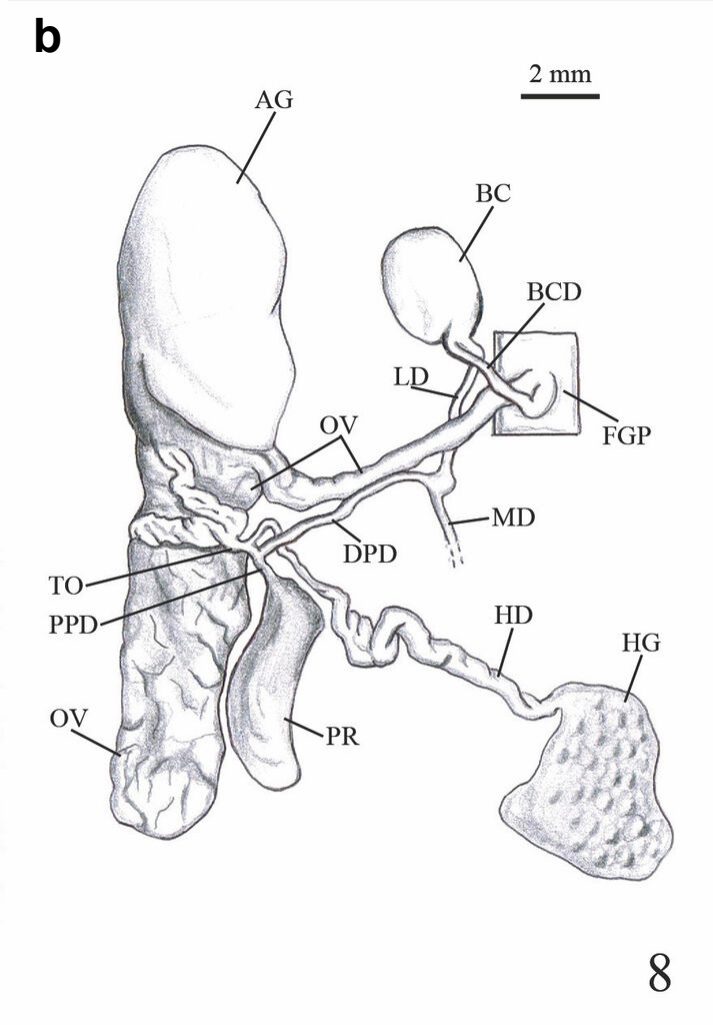
Drawing of the female reproductive system.
